# Clinical Relevance of Antibiotic Susceptibility Profiles for Screening Gram-negative Microorganisms Resistant to Beta-Lactam Antibiotics

**DOI:** 10.3390/microorganisms8101555

**Published:** 2020-10-09

**Authors:** Francisco Montiel-Riquelme, Elisabeth Calatrava-Hernández, Miguel Gutiérrez-Soto, Manuela Expósito-Ruiz, José María Navarro-Marí, José Gutiérrez-Fernández

**Affiliations:** 1 Department of Microbiology, School of Medicine, University of Granada-ibs, 18012 Granada, Spain; fmontiel@correo.ugr.es; 2 Department of Microbiology, Hospital Universitario Virgen de las Nieves-ibs, 18012 Granada, Spain; elisabeth.calatrava.sspa@juntadeandalucia.es (E.C.-H.); josem.navarro.sspa@juntadeandalucia.es (J.M.N.-M.); 3 Department of Emergency, Hospital de la Agencia Sanitaria Alto Guadalquivir, 14550 Montilla, Spain; mgutierrezs@epag.es; 4 Department of Investigation, Hospital Universitario Virgen de las Nieves, 18012 Granada, Spain; manuela.exposito.ruiz@juntadeandalucia.es

**Keywords:** beta-lactam antibiotics, resistance, *Enterobacterales*, *Pseudomonas*, *Acinetobacter*

## Abstract

The increasing resistance to antibiotics is compromising the empirical treatment of infections caused by resistant bacteria. Rapid, efficient, and clinically applicable phenotypic methods are needed for their detection. This study examines the phenotypic behavior of β-lactam-resistant Gram-negative bacteria grown on ChromID ESBL medium with ertapenem, cefoxitin, and cefepime disks, reports on the coloration of colonies, and establishes a halo diameter breakpoint for the detection of carbapenemase-producing bacteria. We studied 186 β-lactam-resistant Gram-negative microorganisms (77 with extended spectrum beta lactamase (ESBL), 97 with carbapenemases, and 12 with AmpC β-lactamases (AmpC)). Susceptibility profiles of Gram-negative bacteria that produced ESBL, AmpC, and carbapenemases were similar to the expected profiles, with some differences in the response to cefepime of ESBL-producing microorganisms. Coloration values did not differ from those described by the manufacturer of ChromID ESBL medium. In the screening of carbapenemase production, inhibition halo diameter breakpoints for antibiotic resistance were 18 mm for *Enterobacterales* and ertapenem, 18 mm for *Pseudomonas* and cefepime, and 16 mm for *Acinetobacter baumannii* and cefepime. This innovative phenotypic approach is highly relevant to clinical laboratories, combining susceptibility profiles with detection by coloration of high-priority resistant microorganisms such as carbapenemase-producing *A. baumannii*, carbapenemase-producing *Pseudomonas* spp., and ESBL and/or carbapenemase-producing *Enterobacterales*.

## 1. Introduction

The increasing resistance to antibiotics is a major worldwide health problem, and the WHO has highlighted the threat posed by extended-spectrum β-lactamase (ESBL)-producing *Enterobacteriaceae* and carbapenemase-producing *Acinetobacter baumannii*, *Pseudomonas aeruginosa,* and *Enterobacteriaceae* [1]. Simple, rapid, effective, and inexpensive techniques are needed to screen for the presence of these pathogens in the digestive tract of infected patients and their contacts [2]. Techniques to detect ESBL-producing enterobacteria colonies include the utilization of CHROMID ESBL (bioMérieux, France), a transparent medium that contains cefpodoxime [3] and other substances that inhibit Gram-positive bacteria growth alongside chromogenic substrates for the presumptive identification of genera and species by their color (pink/burgundy for *Escherichia coli*; blue/green for *Klebsiella*, *Enterobacter*, *Serratia*, or *Citrobacter*; and light to dark brown for *Proteae*) [4]. The inclusion of cefoxitin (FOX), cefepime (FEP), and imipenem disks on CHROMID ESBL medium has been proposed to identify ESBL- and/or carbapenemase-producing microorganisms resistant to these antibiotics, although a halo diameter breakpoint of 15 mm did not prove diagnostically useful [5]. However, the diagnostic performance may be improved by considering a higher halo breakpoint (16 mm) and replacing imipenem with ertapenem (ERT). Carbapenemase or ESBL production is frequently studied in episodes of colonization by multi-resistant Gram-negative bacteria, and the addition of ERT, FOX, and FEP disks to this medium may offer a simple and effective method. ERT has a higher activity against ESBL-producing *Enterobacterales* but a lower activity against carbapenemase-producing *Enterobacterales* in comparison to imipenem [6]. This is why it is used after halo diameter analysis to screen for carbapenemase-producing *Enterobacterales* with or without ESBL. In contrast, *Pseudomonas* spp. and *A. baumannii* have a low intrinsic susceptibility to ERT [6,7], and ERT disks can be used to assess in vitro susceptibility. For its part, FEP has high antimicrobial potency against these bacteria [8] and is useful, after studying the halo diameter for *Pseudomonas* spp. and *A. baumannii,* to discriminate between isolates that produce carbapenemases, the main acquired resistance mechanism, and those that do not.

Tests that use cultures to detect colonies of resistant microorganisms offer an advantage over PCR tests because they detect viable microorganisms. Unfortunately, no commercial culture tests are available to simultaneously detect Gram-negative microorganisms with different mechanisms of resistance to β-lactam antibiotics.

With the above background, the objective of this study was to evaluate the behavior of these microorganisms, which have resistance mechanisms attributed to β-lactamases, in the ChromID^®^ ESBL medium, using the method of antibiogram by diffusion with FOX, CEF, and ERT disks to screen for this resistance. A further aim was to determine the optimal CEF and ERT halo diameters to screen for carbapenemases, which constitute the main resistance mechanism for *Pseudomonas*, *Acinetobacter*, and *Enterobacterales*.

## 2. Material and Methods

### 2.1. Studied Microorganisms 

This retrospective study on the growth of β-lactam-resistant Gram-negative clinical isolates included 186 strains of Gram-negative bacteria with different resistance mechanisms [9] isolated from clinical samples in the Microbiology Department of our hospital in Granada, Spain: 77 with ESBL (17 *Klebsiella pneumonie*, 58 *E. coli* and 2 *Proteus mirabilis*); 97 with the following carbapenemases (Table 1): New Delhi carbapenemase (NDM) (4 *K. pneumoniae*), *Klebsiella pneumoniae* carbapenemase (KPC) (2 *K. pneumoniae*, 1 *Klebsiella oxytoca*, 1 *Citrobacter freundii*), IMP carbapenemase (IMPase) (21 *Pseudomonas* spp., 1 *K. pneumoniae*), Verona integron-encoded metallo-beta-lactamase (VIM) (1 *C. freundii*, 5 *Enterobacter cloacae*, 1 *K. pneumoniae*, 2 *K. oxytoca*, 2 *E. coli,* 7 *Pseudomonas* spp.), and oxacillinase (OXA) (31 *A. baumannii*, 14 *K. pneumoniae*, 2 *E. cloacae*, 2 *E. coli*, 1 *C. freundii*); and 12 with AmpC and FOX-resistance (4 *E. coli*, 2 *K. pneumonie*, 2 *E. cloacae*, 2 *Enterobacter gergoviae*, 1 *Klebsiella aerogenes*, 1 *Morganella morganii*). The MicroScan system (Beckman Coulter, Brea, CA, USA) and mass spectrometry (Maldi-Tof^®^, Bruker Daltonik GmbH, Bremen, Germany) were used to identify isolates. The MicroScan microdilution system was employed to characterize resistance, followed by carbapenemase determination, when appropriate, with the Rapidec^®^ Carba NP colorimetric test (BioMerieux, Marcy l’Etoile, France) and immunochromatography (NG5-Test Carba, NG Biotech, Guipry, France; or OXA-23 K-Set, CorisBioConcept, Gembloux, Belgium). Carbapenemase-producing type was confirmed by the Andalusian Molecular Typing Laboratory of the Spanish PIRASOA Program using mass sequencing (Illumina Inc, San Diego, CA, USA), CLC Genomics Workbench v10 software (Qiagen), and ResFinder (Lyngby, Denmark) (https://cge.cbs.dtu.dk/services/ResFinder) and CARD (Hamilton, ON, Canada) (https://card.mcmaster.ca/) databases.

AmpC production was defined by resistance to FOX and synergy with cloxacillin, and ESBL production was defined by resistance to cefotaxime and/or ceftazidime and synergy with clavulanic acid.

### 2.2. Investigation of Bacteria Growth on CHROMID ESBL Medium

An 0.5 McFarland suspension of each isolate was prepared from colonies grown on lamb blood agar (Becton Dikinson, Franklin Lakes, NJ, USA). Next, a sterilized swab was soaked with the homogenized suspension, excess liquid was removed, and it was uniformly seeded on one half of the plate, streaking the bacterial load on the other half with a calibrated inoculation loop. FEP (30 µg, Becton Dickinson), FOX (30 µg, Becton Dickinson), and ERT (10 µg, Becton Dickinson) disks were then placed equidistantly on the seeded area. The medium was then incubated for 48 h at 37 °C, with readings at 24 h. The isolate was considered susceptible if the inhibition halo was ≥1.6 cm at 24 h and resistant if it was <1.6 cm. We also analyzed CEF-generated inhibition halo diameters to detect carbapenemase-producing *A. baumannii* and *Pseudomonas* and ERT-generated diameters to detect carbapenemase-producing *Enterobacterales*. The optimal breakpoint was evaluated by constructing a receiver operating characteristic (ROC) curve based on the halo diameters. 

### 2.3. Statistical Analysis 

The Wilcoxon test was used to analyze the inhibition halo results for isolates with ESBL. The Kruskal–Wallis or Mann–Whitney U test was used for comparative analysis of carbapenemase enzymatic activity, comparing the inhibition halo diameters generated by the isolate. *p* ≤ 0.05 was considered significant. The breakpoint of the CEF and ERT halo that best defined the presence of carbapenemase was determined according to the area under the receiver operating characteristic curve [10], after calculating the optimal breakpoints using the Youden index (sum of sensitivity and specificity minus one). IBM SPSS Statistics 19 and Microsoft Excel 2019 were used for statistical analyses.

## 3. Results

### 3.1. Behavior of Susceptibility Profiles with CEF, FOX, and ERT Disks

#### 3.1.1. *Enterobacterales* with ESBL

**Figure 1:**
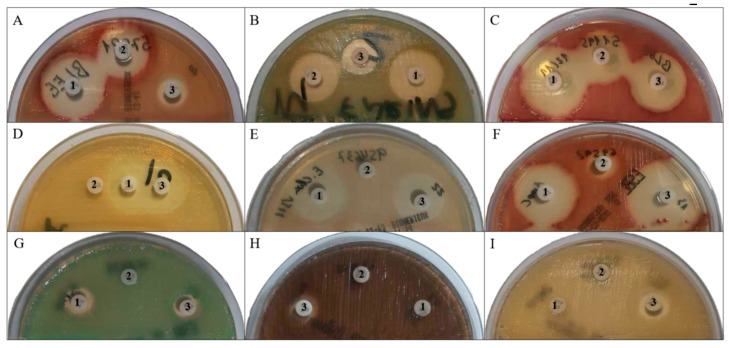
Coloring and susceptibility profile of the different species studied on CHROMID extended spectrum beta lactamase (ESBL) plates. (**A**) *E. coli* ESBL; (**B**) *K. pneumoniae* ESBL; (**C**) susceptible to cefepime *E. coli* ESBL; (**D**) *E. gergoviae* AmpC; (**E**) *E. cloacae* AmpC; (**F**) *E. coli* AmpC; (**G**) carbapenemase-producing *K. pneumoniae*; (**H**) carbapenemase-producing *P. aeruginosa*; (**I**) carbapenemase-producing *A. baumannii*. Antibiotic disks are marked 1 for ertapenem, 2 for cefoxitin and 3 for cefepime.

Table 2 and Figure 1 display the color distribution of microorganisms. Although all isolates were resistant to FEP in microdilution, the inhibition halos varied widely (Table 3) and were almost always lower for susceptible isolates (34; 44.15%) than for halos with FOX (31; 91.18%) (*p* = 0.00). Isolates were almost always (74; 96.1%) susceptible to FOX (Table 3), and all isolates were susceptible to ERT (halos >20 mm). Hence, almost all isolates (96.1%) were susceptible to both ERT and FOX, and showed a variable response to FEP.

#### 3.1.2. *Enterobacterales* with AmpC

Table 2 and Figure 1 exhibit the color distribution of the microorganisms. All isolates were susceptible to ERT and FEP (halos >19 mm.) and almost all were resistant to FOX (11; 91.7%), except for one *E. gergoviae* isolate (halo = 17 mm, which was resistant by microdilution (MIC >16 mg/L). This isolate was also resistant to amoxicillin/clavulanic acid. Hence, almost all (91.7%) of the isolates were susceptible to ERT and FEP and resistant to FOX.

#### 3.1.3. Carbapenemase-producing microorganisms 

**Figure 2:**
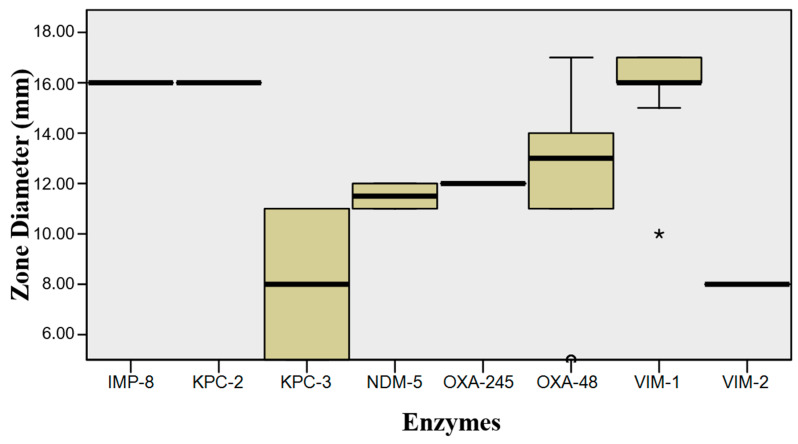
Distribution of diameters produced in *Enterobacterales* by each enzyme with ertapenem disc.

**Figure 3:**
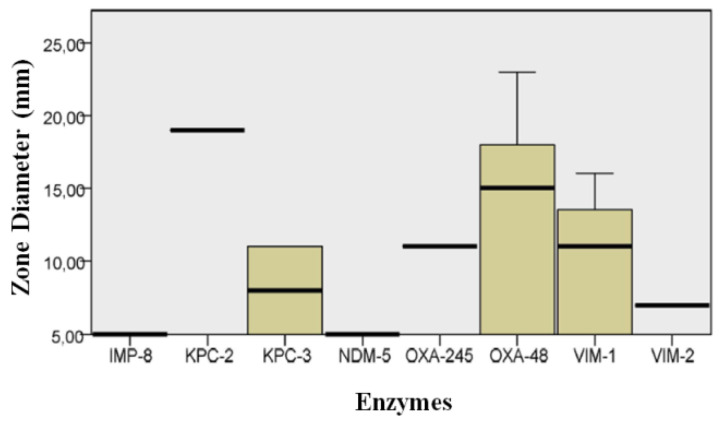
Distribution of diameters produced in Enterobacterales by each enzyme with cefoxitin disc.

These included *Enterobacterales*, *Pseudomonas*, and *A. baumannii*. Table 2 and Figure 1 show the color distribution of *Enterobacterales*. *K. oxytoca* had a lighter green-blue shade in comparison to *K. pneumoniae*. *E. cloacae* had a green-blue shade of varying intensity, although some colonies were beige. *C. freundii* and *E. coli* showed a similar color pattern, with some pink and other light beige isolates, although in different percentages. Disk results were as follows: 8 (21%) isolates were susceptible to ERT, with halos between 16 and 17 mm that were larger in the presence of VIM-1 and smaller in the presence of KPC-3 (*p* = 0.022) (Figure 2); 10 (26%) were susceptible to FOX, with halos between 16 and 23 mm (*p* = 0.193) (Figure 3); and 7 (18%) were susceptible to FEP, with halos ≤18 mm (*p* = 0.289). Hence, almost all (94.7%) of the 36 isolates were resistant to ERT and/or FEP.

Table 2 and Figure 1 show the color distribution of the *Pseudomonas*. No halos were observed with ERT or FOX for any isolate and the halo was always <16 mm with FEP, being larger in the presence of VIM-2 (*p* = 0.032).

In ChromID ESBL medium, 28 (90.3%) of *A. baumannii* colonies were light beige and 3 (9.7%) were beige (Table 2 and Figure 1). All isolates were resistant to all three antibiotics, although halos <15 mm were generated with FEP, with no difference according to the type of OXA (*p* = n.s.).

### 3.2. Calculation of the Breakpoint with ERT and FEP for Carbapenemase-producing Microorganisms 

#### 3.2.1. Enterobacterales and ERT

**Figure 4:**
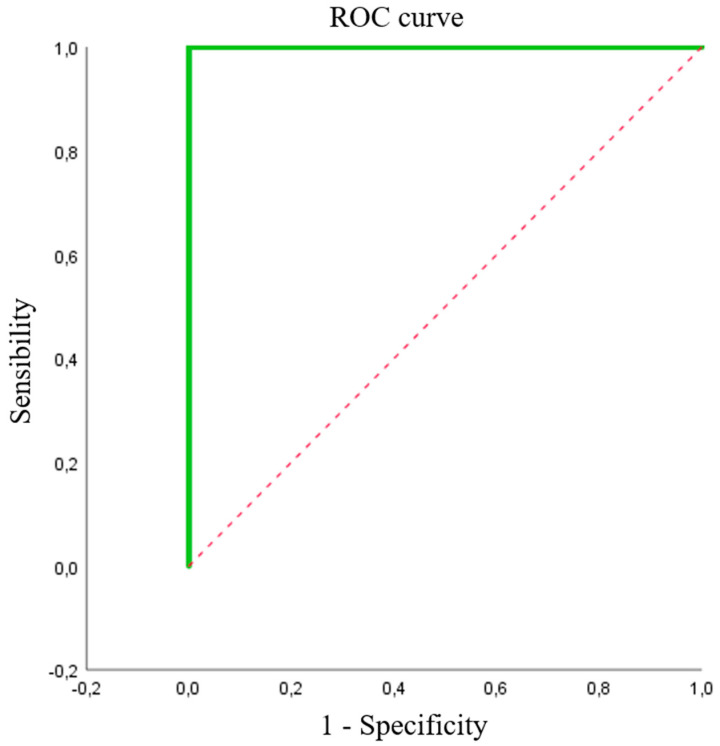
ROC curve calculated for *Enterobacterales*. The ROC curve is green and the reference diagonal line red.

We studied 115 isolates: 38 (35%) were carbapenemase-producing, with halos between 5 and 17 mm (11.69 ± 3.704) for ERT, and 77 (65%) were ESBL-producing, with halos between 19 and 37 mm (27.06 ± 3.290) for ERT, as control group. The optimal breakpoint calculated using Youden’s index was 18 mm, with a sensitivity of 100% and specificity of 100% and area under the ROC curve of 1 (Figure 4).

#### 3.2.2. Pseudomonas spp. and CEF

**Figure 5:**
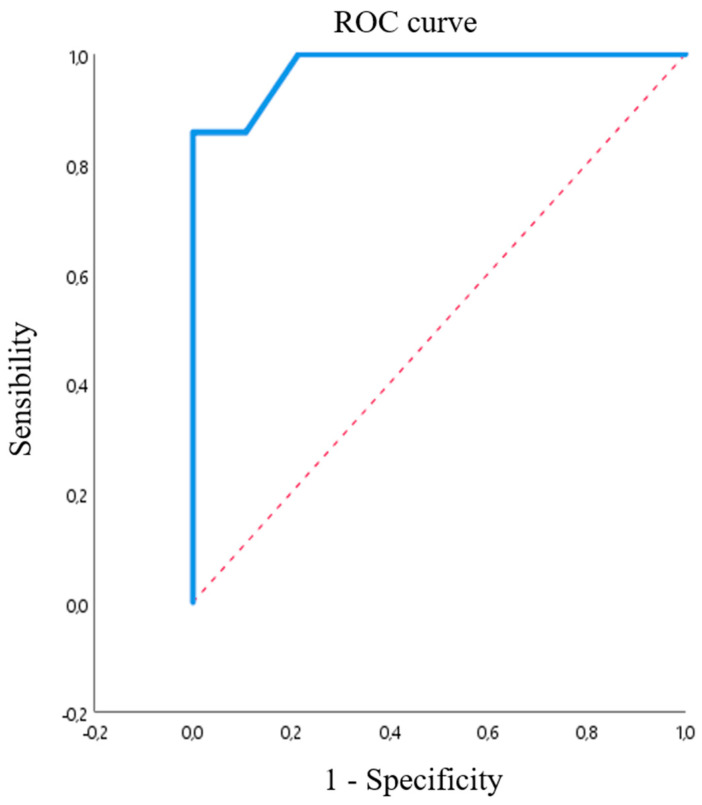
ROC curve calculated for *P. aeruginosa.* The ROC curve is blue, and the reference diagonal line is red.

We studied 42 isolates: 28 (66.7%) were carbapenemase-producing, with halos between 5 and 15 mm (10.75 ± 3.28) for FEP, and 14 (33.3%) were non-carbapenemase-producing *P. aeruginosa*, with halos between 14 and 37 mm for FEP (24.43 ± 6.63), as control group. The optimal breakpoint calculated using Youden’s index was 18 mm, with a sensitivity of 100% and specificity of 97.7% and area under the ROC curve of 0.977 (Figure 5). 

#### 3.2.3. *A. baumannii* and CEF

**Figure 6:**
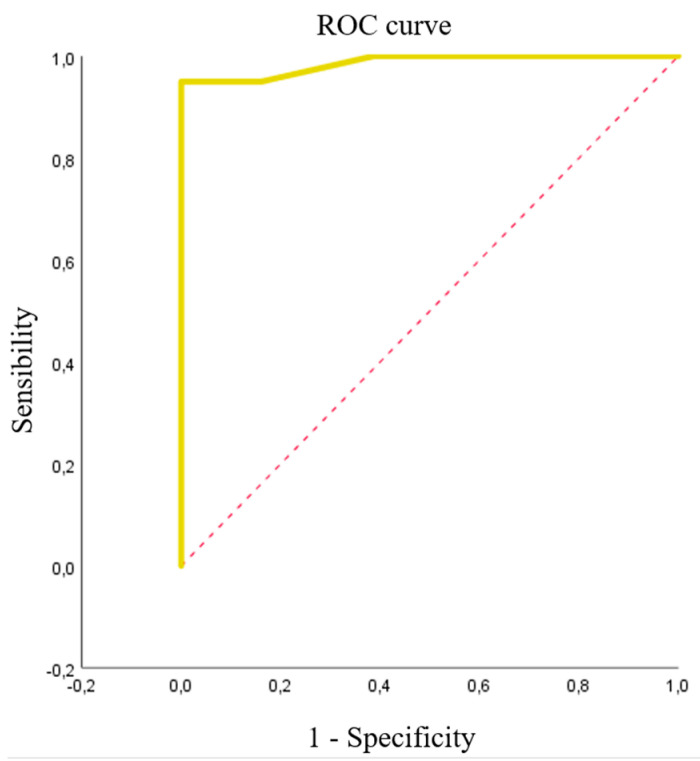
ROC curve calculated for *A. baumannii.* The ROC curve is yellow, and the reference diagonal line is red.

We studied 71 isolates: 31 (43.7%) were carbapenemase-producing *A.baumannii*, with halos between 5 and 15 mm (12.45 ± 2.142) for FEP, and 40 were non-carbapenemase-producing microorganisms (2 *A. baumannii,* 14 *P. aeruginosa,* and 24 *Enterobacterales*), with halos between 14 and 39 mm for FEP (29.61 ± 6.727), as control group. The optimal breakpoint calculated using Youden’s index was 16 mm, with a sensitivity of 100% and specificity of 95% and area under the ROC curve of 0.986 (Figure 6).

## 4. Discussion

### 4.1. Susceptibility Profiles with CEF, FOX, and ERT Disks

The expected susceptibility profile of ESBL-producing microorganisms shows resistance to FEP and susceptibility to FOX and ERT in antibiograms based on microdilution. All studied isolates were susceptible to ERT, reflecting the fact that carbapenems are frequently the treatment of choice against infections produced by these bacteria due to the low capacity of ESBLs to modify these molecules [11,12]. Most of the isolates were susceptible to FOX but three were resistant, which may be associated with membrane permeability reduction phenomena observed in some ESBL-producing bacteria [13], given that they were susceptible to amoxicillin/clavulanic acid and piperacillin/tazobactam. Although FOX has been proposed to treat some infections caused by ESBL-producing bacteria [14], its use must be highly controlled, because a decrease in porin number and/or expression of AmpC-type enzymes is frequently observed during the treatment [15,16].

Reports on the in vitro effect of FEP against Enterobacterales are highly varied, and numerous *E. coli* isolates have been found to be susceptible to this antimicrobial [17]. FEP can be clinically active in some cases, although it is not recommended for infections caused by ESBL-producing bacteria because of the high failure rate [18,19,20]. 

In our series, the response to FEP of ESBL-producing isolates was also varied, with 55.8% being resistant to FEP disks when cultured in plate, contrasting with the results obtained with microdilution, when all isolates were resistant with a MIC value >8 mg/L. This discrepancy may have various explanations: 1) the lowering of the breakpoint by the CLSI in 2014 [21]; 2) reports on isolates susceptible to FEP and with ESBL from the SHV family, which are less active against this antibiotic than CTX-M-type β-lactamases [15,22]; and 3) a possible synergic effect between the FEP disk used in this study and the cefpodoxime in the ChromID ESBL medium, leading to the classification of numerous isolates as susceptible. The combination of both antibiotics may saturate β-lactamases, most of which have enzymatic kinetics that follow the Michaelis–Menten equation [23]. However, given that FEP and cefpodoxime will not be used in combination in the clinical setting, classification of the strains based on the microdilution method yields more relevant information. In addition, the Wilcoxon test confirmed that most isolates susceptible to FEP had a smaller halo in comparison to the FOX halos. This result is novel and can be applied in clinical diagnosis.

The expression of class C β-lactamases, such as AmpC, is constitutive and inducible in microorganisms of the *Enterobacter and Citrobacter* genera [24] under study here. The remaining isolates belonged to *E. coli* and *K. pneumonia* species. They do not constitutively express these types of enzyme because they lack the regulating genes, and their expression is produced by the acquisition of plasmids from the chromosomal β-lactamases of *E. cloacae* (ACT-1 and MIR-1), *C. freundii* (CMY), *Hafnia alvei* (ACC-1), and *M. morganii* (DHA-1) [25]. The observation of CMY-2 and DHA-1 in *E. coli* isolates and DHA-1 in *K. pneumoniae* isolates is very frequent [26]. The expected profile for AmpC-producing isolates is susceptibility to ERT and FEP and resistance to FOX, and the present isolates were always susceptible to ERT and FEP. The expression of AmpC-type enzymes does not usually confer resistance to carbapenems or 4^th^ generation cephalosporins [27] except when the overexpression of these β-lactamases is combined with a reduction in porin number [28,29], when there may be a significant increase in the MIC value of carbapenems such as ERT or imipenem. One isolate was susceptible to FOX in the ChromID ESBL medium but showed resistance in the antibiogram based on microdilution. This is unusual, because FOX is the main antibiotic used to detect this type of resistance. The diameter of the halo for this isolate was 17 mm, suggesting the need to raise the breakpoint used to detect this type of resistance. Research in more isolates is also required.

In relation to our study of carbapenemase-producing Enterobacterales, we highlight that they included a large number of *K. pneumoniae* isolates (57.9%), mainly carriers of β-lactamase OXA-48. This is attributable to the frequency of this enzyme in *K. pneumoniae,* which produces endemic infections in North African countries [30] and nosocomial outbreaks in Spain, among other countries [31,32]. Other *E. coli* and *C. freundii* isolates also showed this type of enzyme. Other types of β-lactamase frequently encountered in isolates of Enterobacterales include metallo-β-lactamases, such as the VIM present in *E. cloacae*, *E. coli*, *K. oxytoca*, and *C. freundii,* among other species. These isolates expressed VIM-1-type enzymes, whereas these Enterobacterales have generally been found to express VIM-2-type enzymes [33]. We also highlight the study of isolates of *K. pneumoniae* with NDM, which has shown a rapid expansion over the past few years [34].

There have been reports on the limited activity of OXA-48-type enzymes, which have low degradation capacity against extended-spectrum cephalosporins such as ceftazidime and carbapenems. For this reason, isolates expressing this enzyme that are identified as resistant to carbapenems frequently present other associated mechanisms, e.g., simultaneous ESBL expression and porin decrease [16,35]. The low degradation activity of OXA-48-type enzymes can lead to the clinical non-detection of its presence in some cases, thereby contributing to the spread of these isolates.

Our statistical analysis revealed that halo diameters were significantly larger for VIM-1- versus OXA-48-carrier bacteria on ERT disks, which may indicate a lower activity for this type of enzyme than for OXA-48 carbapenemases. Although the difference was not significant, the diameter range generated by bacteria expressing the KPC-3 enzyme was also lower, as in the case of the OXA-48 enzyme, although the isolated activity of KPC-3 was not sufficient to generate resistance to all β-lactams [36,37,38].

In the case of FOX, no significant difference was found between isolates with VIM-1 and OXA-48 enzymes, which showed a wide range of values that indicated their variable activity. OXA-48 halos had a large mean diameter, indicating the low activity of this enzyme. Once more, KPC-3-producing isolates showed small or absent halos. The reduced number of isolates expressing the remaining enzymes hampers further statistical analyses. In the case of FEP, the halo range did not significantly differ among all enzymes, observing a wide variability in KPC-3 halos and a larger diameter range for VIM-1 and VIM-2 enzymes. The lack of statistical significance may be attributable to the small number of isolates for each type of carbapenemase. As expected, the susceptibility profile study evidences general resistance to all three antibiotics under study; although we highlight resistance to ERT and/or FEP as a screening marker. The emergence of isolates susceptible to these antimicrobials may be explained by the finding that the isolated activity of carbapenemases in vitro is insufficient to produce a resistance profile [11,36] and indicates the need to establish a breakpoint >16 mm for screening.

The carbapenemase-producing *Pseudomonas* were largely *P. aeruginosa*, which expressed metallo-β-lactamase-type carbapenemases, mainly type IMP. The combination of carbapenemases with other resistance mechanisms, mainly porin underexpression and AmpC overexpression [39], create high resistance to most β-lactam antibiotics, explaining the profile obtained in this study, in which 100% of isolates were resistant to ERT, FEP, and FOX. A significantly larger halo diameter was observed when VIM-2 was present, suggesting its lower activity, although these data should be interpreted with caution given the low number of isolates. No significant differences were found for the other enzyme groups. The resistance profile observed for these carbapenemase-producing *Pseudomonas* underscores the critical clinical importance of these microorganisms, because their presence (frequent in intensive care units) requires the use of antibiotics of last resort, e.g., colistin [40,41].

Isolates of *A. baumannii* only presented OXA-type class D β-lactamases, often observed in isolates of this species [35]. Their presence is increasingly implicated in the etiology of nosocomial infections due to the expression of carbapenemases, mainly OXA-23, followed by OXA-58, alongside their abundant intrinsic resistance mechanisms [41,42]. The expression of these two enzymes has previously been reported [43]. These carbapenemases and the other resistance mechanisms in *A. baumannii* confer a similar resistance profile to that of *Pseudomonas*, which showed no inhibition halos with ERT or FOX antibiotics and halos <1**5** mm with FEP, identified as resistance by the screening test using ChromID ESBL medium. Comparative study of the halo diameters showed no significant differences between the halos produced by microorganisms expressing the two aforementioned enzymes, suggesting that the two carbapenemases exert a similar activity.

According to the manufacturer of ChromID ESBL medium, colonies of ESBL-producing *E.coli* are identified by their pink color, colonies of ESBL-producing *Klebsiella* spp., *Enterobacter* spp., *Serratia* spp., and *Citrobacter* spp. by their green-blue color, and those of the *Proteae* tribe (*Proteus* spp., *Morganella* spp. and *Providencia* spp.) by their dark to light brown color. *Pseudomonas* and *Acinetobacter* genera are not included in the manufacturer’s list. The above colors were shown by most isolates in this study, although we highlight the white color of some isolates of *E. coli* (2), *C. freundii* (1), and *E. cloacae* (3). A light green-blue color was observed for *K. oxytoca* isolates and *E. cloacae* and *E. aerogenes* colonies, as reported by Romo-Ibáñez et al., 2020. The brown or beige color of isolates of the *Pseudomonas* genus is attributed by the manufacturer to the *Proteae* tribe, although they can be readily differentiated by the oxidase test. Most *A. baumannii* colonies were light beige, occasionally turning into cream beige, hampering their differentiation from some light beige Enterobacterales isolates.

Selective chromogenic media such as ChromID ESBL may facilitate work in clinical diagnosis laboratories by allowing the presumptive identification of species or groups of microorganisms, alongside their generic resistance profile, which was to cefpodoxime in the present case. If the aforementioned antibiotic disks are added to this medium, a screening strategy can be implemented to detect β-lactam resistant microorganisms.

### 4.2. Breakpoint Calculation with ERT and FEP for the Screening of Carbapenemase-producing Microorganisms

In previous studies [10], construction of a ROC curve was effective to determine a breakpoint that differentiated between isolates resistant and susceptible to certain antibiotics in media such as MacConkey agar. However, this medium does not allow the differentiation of Gram-negative microorganisms present in a clinical sample and does not inhibit the growth of susceptible isolates. Nonetheless, the MacConkey agar medium is less expensive than the ChromID ESBL medium. In our study, ROC curves were constructed for Enterobacterales and carbapenemase-producing non-fermenting Gram-negative bacilli in ChromID ESBL medium with ERT and FEP disks, respectively.

For carbapenemase-producing Enterobacterales, the area under the curve was 1 and sensitivity and specificity values were 100% when considering a breakpoint halo diameter of 18 mm, likely attributable to the wide difference between the halos of non-carbapenemase-producing and carbapenemase-producing microorganisms. In other words, this test perfectly discriminated between producing and non-producing isolates, although it would be desirable to study a wider range of carbapenemases.

For *Pseudomonas*, the area under the ROC curve was 0.977, the sensitivity was 100%, and the specificity was 85.7% for a breakpoint of 18 mm. As above, these data should be interpreted with caution due to the reduced number of isolates.

For *A. baumannii,* a different bacterial pool had to be used to study halos produced with FEP, because of the difficulty in finding *A. baumannii* susceptible to β-lactams in our setting. The area under the ROC curve was 0.986 for a breakpoint halo diameter of 16 min, which yielded sensitivity of 100% and specificity of 95%. In other words, as with the breakpoint for *Pseudomonas,* there is a high likelihood of correctly evaluating isolates as resistant when the halo is below the breakpoint and as susceptible when the halo is equal to or larger than the breakpoint diameter. Besides the small number of resistant isolates, a further limitation of the test was the use of bacteria other than *A. baumannii* as negative control group.

### 4.3. Study Limitations for Its Application in Clinical Practice

The combined presence in a clinical isolate of different resistance mechanisms (porin decrease, β-lactamases,…) generates different antibiotic susceptibility profiles for each microorganism, and the presence of carbapenemases needs to be detected by using multiple phenotypic tests, such as a combination of antibiogram screening by diffusion, placing disks on ChromID ESBL medium, with an immunochromatography test. However, molecular techniques are required to confirm the expression of carbapenemases and identify resistance genes, given the clinical relevance of this information in hospitals. Further studies are needed in a larger number of isolates to confirm these preliminary results and the value of the ESBL medium with disks for screening clinical samples with ESBL- and Carbapenemase-mediated resistances.

## 5. Conclusions

Microbiology laboratories should provide economic and easily-applied screening tests to detect microorganisms resistant to β-lactams, given their increasing clinical importance. This study highlights the usefulness of ChromID ESBL medium with the placement of antibiotic disks to screen for resistant Gram-negative microorganisms when inhibition haloes are <16 mm. In this way, ESBL-producing Enterobacterales are susceptible to ERT and FOX disks, and AmpC producers are resistant to FOX and susceptible to ERT and FEP. Most carbapenemase producers are resistant to ERT and/or FEP, although a better diagnostic performance was achieved with ERT and a halo breakpoint of 18 mm. Carbapenemase-producing *A. baumannii* and *Pseudomonas* spp. are resistant to ERT, FOX, and FEP, although an excellent diagnostic performance was obtained for *Pseudomonas* spp. with FEP and a halo breakpoint of 18 mm. Alongside the differential coloration of colonies, this approach permits the implementation of a simple and fast method to screen for resistant microorganisms in clinical microbiology laboratories, allowing focus on the search for a specific resistance mechanism in detected microorganisms.

## Figures and Tables

**Table 1:** Types of carbapenemases in *Enterobacterales*, *Pseudomonas*, and *Acinetobacter*, performed by the Andalusian Molecular Typing Laboratory.

				Types of Carbapenemases												Total
				VIM-		KPC-		NDM-5	IMP-			OXA				
				−1	−2	−2	−3		−8	−16	−23	−48	−23	−58	−245	
*Enterobacterales*		*Escherichia coli*		2	0	0	0	0	0	0	0	2	0	0	0	4
		*Enterobacter cloacae*		5	0	0	0	0	0	0	0	2	0	0	0	7
		*Klebsiella oxytoca*		2	0	1	0	0	0	0	0	0	0	0	0	3
		*Klebsiella pneumoniae*		1	0	0	2	4	1	0	0	13	0	0	1	22
		*Citrobacter freundii ^a^*		0	1	1	0	0	0	0	0	1	0	0	0	3 ^a^
*Pseudomonas*		*P. aeruginosa*		2	3	0	0	0	12	7	2	0	0	0	0	26
		*P. putida*		1	1	0	0	0	0	0	0	0	0	0	0	2
*Acinetobacter*		*A. baumannii*		0	0	0	0	0	0	0	0	0	19	12	0	31
**Total**				13	5	2	2	4	13	7	2	18	19	12	1	98

**^a^** One isolate included VIM-2 and KPC-2 enzymes.

**Table 2:** Distribution of isolates with ESBL according to the color of colonies on ChromID ESBL medium.

Color of Colonies on ESBL								
Microorganisms	Light Beige	Green-Blue	Light Green-Blue	Pink	Light Pink	Light Brown	Brown	Total
*E. coli* ESBL	1	0	0	54	3	0	0	58
*E. coli* AmpC	0	0	0	4	0	0	0	4
*E. coli* CAR	1	0	0	3	0	0	0	4
*K. pneumoniae* ESBL	0	17	0	0	0	0	0	17
*K. pneumoniae* AmpC	0	1	1	0	0	0	0	2
*K. pneumoniae* CAR	0	22	0	0	0	0	0	22
*P. mirabilis* ESBL	0	0	0	0	0	2	0	2
*C. freundii* AmpC	1	0	0	0	0	0	0	1
*C. freundii* CAR	1	0	0	1	0	0	0	2
*K. aerogenes* AmpC	0	1	0	0	0	0	0	1
*E. cloacae* AmpC	0	0	2	0	0	0	0	2
*E. cloacae* CAR	3	3	1	0	0	0	0	7
*E. gergoviae* AmpC	0	0	2	0	0	0	0	2
*K. oxytoca* CAR	0	0	3	0	0	0	0	3
*P. aeruginosa* CAR	14	0	0	0	0	0	12	26
*P. putida* CAR	2	0	0	0	0	0	0	2
*A. baumannii* CAR	31	0	0	0	0	0	0	31
**Total**	54	44	9	62	3	2	12	186

CAR: carbapenemase producer.

**Table 3:** Distribution of isolates with ESBL on ChromID ESBL medium and behavior (%) with cefepime and cefoxitin disks.

	Cefepime		Cefoxitin		Total
	Susceptible	Resistant	Susceptible	Resistant	
*E. coli*	29 (37.7)	29 (37.7)	57 (74.0)	1 * (1.3)	58
*K. pneumoniae*	3 (3.9)	14 (18.2)	15 (19.5)	2 ** (2.6)	17
*P. mirabilis*	2 (2.6)	0 (0)	2 (2.6)	0 (0)	2
**Total**	34 (44.2)	43 (55.8)	74 (96.1)	3 (3.9)	77

Halos of * 14 and ** 13/14 mm with MIC = 16 mg/L and susceptible to amoxicillin/clavulanic and piperacillin/tazobactam by microdilution.
